# The expression of mimecan in adrenal tissue plays a role in an organism’s responses to stress

**DOI:** 10.18632/aging.202991

**Published:** 2021-05-10

**Authors:** Bin Su, Qian-Yue Zhang, Xue-Song Li, Hui-Min Yu, Ping Li, Jun-Hua Ma, Huang-Ming Cao, Fei Sun, Shuang-Xia Zhao, Cui-Xia Zheng, Ying Ru, Huai-Dong Song

**Affiliations:** 1 Department of Blood Transfusion and Endocrinology, Shanghai Tenth People’s Hospital, Tongji University School of Medicine, Shanghai 200072, China; 2 The Core Laboratory in Medical Center of Clinical Research, Department of Molecular Diagnostic and Endocrinology, Shanghai Ninth People’s Hospital, State Key Laboratory of Medical Genomics, Shanghai Jiao tong University School of Medicine, Shanghai 200011, China; 3 Department of Endocrine Metabolism, Minhang Hospital, Fudan University, Shanghai 201199, China; 4 Department of Respiration, Yangpu Hospital, Tongji University, Shanghai 200090, China; 5 Department of Endocrinology and Metabolism, Anhui Provincial Hospital, The First Affiliated Hospital of University of Science and Technology of China, Hefei 230001, Anhui, China; 6 Department of Genetics and Genomic Sciences, Icahn School of Medicine at Mount Sinai, New York, NY 10029, USA

**Keywords:** mimecan, stress response, hypothalamic-pituitary-adrenal axis, adrenal gland

## Abstract

Mimecan encodes a secretory protein that is secreted into the human serum as two mature proteins with molecular masses of 25 and 12 kDa. We found 12-kDa mimecan to be a novel satiety hormone mediated by the upregulation of the expression of interleukin (IL)-1β and IL-6 in the hypothalamus. Mimecan was found to be expressed in human pituitary corticotroph cells and was up-regulated by glucocorticoids, while the secretion of adrenocorticotropic hormone (ACTH) in pituitary corticotroph AtT-20 cells was induced by mimecan. However, the effects of mimecan in adrenal tissue on the hypothalamic–pituitary–adrenal (HPA) axis functions remain unknown. We demonstrated that the expression of mimecan in adrenal tissues is significantly downregulated by hypoglycemia and scalded stress. It was down-regulated by ACTH, but upregulated by glucocorticoids through *in vivo* and *in vitro* studies. We further found that 12-kDa mimecan fused protein increased the corticosterone secretion of adrenal cells *in vivo* and *in vitro*. Interestingly, compared to litter-mate mice, the diurnal rhythm of corticosterone secretion was disrupted under basal conditions, and the response to restraint stress was stronger in mimecan knockout mice. These findings suggest that mimecan stimulates corticosterone secretion in the adrenal tissues under basal conditions; however, the down-regulated expression of mimecan by increased ACTH secretion after stress in adrenal tissues might play a role in maintaining the homeostasis of an organism’s responses to stress.

## INTRODUCTION

Each organism must maintain a complex dynamic equilibrium, otherwise known as homeostasis. Stress is a state in which homeostasis is threatened by emotional or physical stressors, although various physiological and behavioral adaptive responses may be restorative in this regard [1]. Exposure to stressful challenges incites behavioral and physical changes that are normally adaptive and limited over time, improving an organism’s chance of survival. These responses must be appropriate in magnitude and duration; otherwise, they may have detrimental effects on numerous physiological functions in an organism, leading to a state of disease-causing disturbed homeostasis.

The hypothalamic–pituitary–adrenal (HPA) axis is pivotal in the response to stress. HPA axis activation is initiated by the activation of parvocellular neurons in the paraventricular nucleus (PVN) of the hypothalamus and the release of corticotropin-releasing hormone (CRH) and arginine vasopressin (AVP) from the external zone of the median eminence into the portal circulation [2, 3]. CRH acts upon the anterior pituitary, stimulating the synthesis and secretion of adrenocorticotropic hormone (ACTH), which then stimulates the adrenal cortex to secrete glucocorticoids (GC) (cortisol in humans; corticosterone in rodents) [2, 3]. A primary effect of stress-induced glucocorticoid release is the inhibition of ongoing HPA axis activation through negative feedback at the level of the hypothalamus and pituitary and at upstream limbic structures, thus inhibiting ACTH and CRH secretion through mineralocorticoid (MR) and glucocorticoid receptors (GR) [2, 3].

HPA-axis dysfunction is implicated in the pathogenesis of various stress-related physical and psychological diseases, such as Cushing’ syndrome, panic disorder, and post-traumatic stress disorder, all of which reflect heightened HPA-axis activity. On the other hand, adrenal insufficiency, chronic fatigue syndrome, and atypical depression are associated with reduced activity of the HPA axis [4, 5]. Hence, mechanisms regulating the functions of the HPA axis are of utmost importance for the management of stress-related disease. Such mechanisms unfortunately have yet to be fully clarified.

Originally isolated from bone, mimecan (osteoglycin) belongs to the family of small leucine-rich proteoglycans (SLRPs) [6]. SLRPs are abundant in the bone matrix, cartilage cells, and connective tissues. They are also essential for collagen fibrillogenesis and are central for the control of cellular growth, differentiation, and migration [7]. Although the mimecan gene encodes a 34-kDa full-length protein, a 12-kDa mature protein corresponding to the 105 carboxyl-terminal amino acids of mimecan has been isolated from bovine bone. A 25-kDa keratan sulfate glycoprotein corresponding to the 223 carboxyl-terminal amino acids of mimecan has been isolated from bovine cornea [8].

Currently, the physiological functions of mimecan remain obscure. We previously cloned the full-length cDNA of human mimecan (accession number: AF100758) [9], establishing mimecan as a novel satiety hormone in adipose tissue propagated by the induction of IL-1β and IL-6 within the hypothalamus [10]. Our previous studies also showed that mimecan is expressed in human pituitary corticotroph cells and the AtT-20 mouse corticotroph cell line, and mimecan gene expression in pituitary corticotroph cells is up-regulated by glucocorticoids (GCs) in a time- and dose-dependent manner [9, 11]. In addition, mimecan stimulates adrenocorticotrophic hormone (ACTH) secretion in pituitary corticotroph AtT-20 cells [12]. Because mimecan is important for the functioning of the HPA axis, the present study was undertaken to further explore the expression of mimecan in the adrenal glands and its effects on HPA axis functions.

## RESULTS

### Mimecan is mainly expressed in the adrenal cortex and medullary mesenchyme

**Figure 1:**
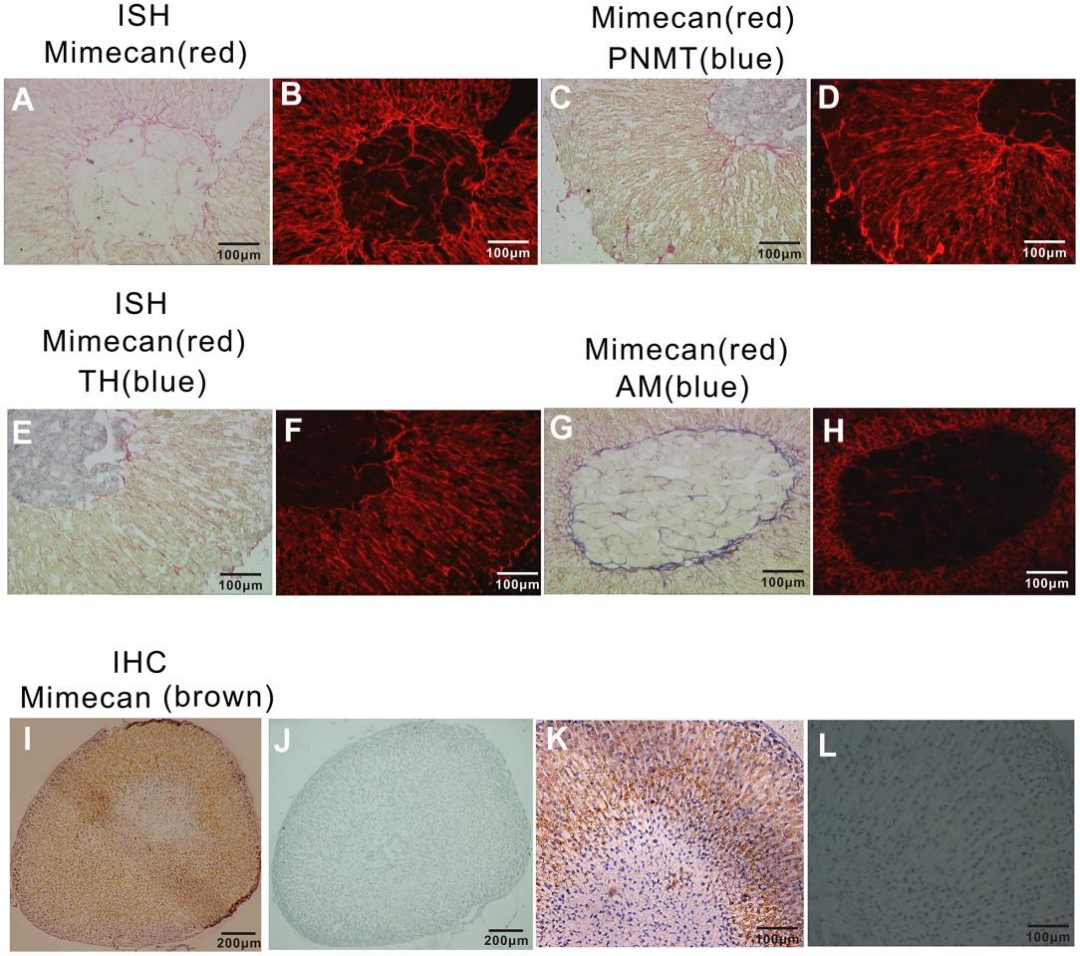
**Localization of mimecan expression in mouse adrenal glands using *in situ* hybridization and immunohistochemistry.** (**A**) Positive staining (red) of sections of adrenal glands hybridized with DIG-labeled mimecan antisense probes primarily appeared in the adrenal cortex and some of the medullary mesenchyme (image obtained under visible light at 10× magnification). (**B**) The field shown is the same as (**A**) under fluorescent light. (**C**) Sections of adrenal glands hybridized with fluorescein-labeled mimecan and DIG-labeled PNMT antisense probes, confirming mimecan expression (red) primarily within the adrenal cortex and PNMT (phenylethanolamine-N methyl transferase; blue) expression in the medulla; no co-expression was observed (image obtained under visible light at 10× magnification). (**D**) The field shown is the same as (**C**) under fluorescent light. (**E**) Sections of adrenal glands hybridized with fluorescein-labeled mimecan and DIG-labeled TH antisense probes, confirming mimecan expression (red) primarily within the adrenal cortex, while TH (blue) was found in the medulla; no co-expression was observed (image obtained under visible light at 10× magnification). (**F**) The field shown is the same as (**E**) under fluorescent light. (**G**) Sections of adrenal glands hybridized with fluorescein-labeled mimecan and DIG-labeled ADREAM antisense probes, confirming mimecan expression (red) primarily within the adrenal cortex. Mimecan and ADREAM co-expression was observed in the medulla (image obtained under visible light). (**H**) The field shown is the same as (**G**) under fluorescent light. (**I**) Sections of formalin-fixed, paraffin-embedded adrenal glands immunostained with rabbit anti-human mimecan antibody and anti-rabbit IgG antibody. Positive (brown) staining was found primarily in the adrenal cortex. (**J**) Negative control of (**I**) with pre-immune rabbit serum used in lieu of mimecan antibody. (**K**–**L**) Higher magnifications of (**I**, **J**).

In this study, we first examined the distribution of mimecan in the adrenal tissues of the C57BL/6 mouse using immunohistochemistry and *in situ* hybridization. As indicated by the results of polyclonal anti-mimecan immunostaining, mimecan is expressed mainly in the adrenal cortex (Figure 1I, 1L). This is consistent with the results of *in situ* hybridization using fluorescein-labeled mimecan antisense RNA probes (Figure 1A, 1B). Adrenal medullary cells are chiefly composed of adrenaline- and noradrenaline-releasing chromaffin cells, both expressing catecholamine synthesizing enzymes, such as tyrosine hydroxylase (TH) and dopamine β-hydroxylase (DBH). Adrenaline-releasing cells alone harbor phenylethanolamine-N methyl transferase (PNMT), an enzyme that methylates noradrenaline, converting it to adrenaline. Adrenomedullin (AM) is a hormone that is highly expressed in the adrenal medullary mesenchyme. To clarify the occurrence of mimecan expression in the adrenal medulla, we performed dual-color *in situ* hybridization of mimecan and PNMT or TH or AM. Neither PNMT (blue) expression in the adrenaline-releasing cells of the adrenal medulla nor TH (blue) expression in most of the adrenal medulla were co-expressed with mimecan (red) (Figure 1C, 1F). However, AM (blue) and mimecan (red) were found to be co-expressed in the medullary mesenchyme (Figure 1G, 1H). In conclusion, these results reveal that, in the mouse adrenal glands, mimecan is mainly expressed in the cortex, although it is also detected in the medulla.

The adrenal cortex is responsible for the synthesis of glucocorticoids, which are essential for survival under stressful conditions. Given the high level of adrenocortical mimecan expression demonstrated by our previous and present studies [11, 12], we presumed that mimecan may be involved in regulating the functions of the HPA axis during the response to stress.

### Mimecan expression in adrenal tissue decreased after exposure to acute stress

**Figure 2:**
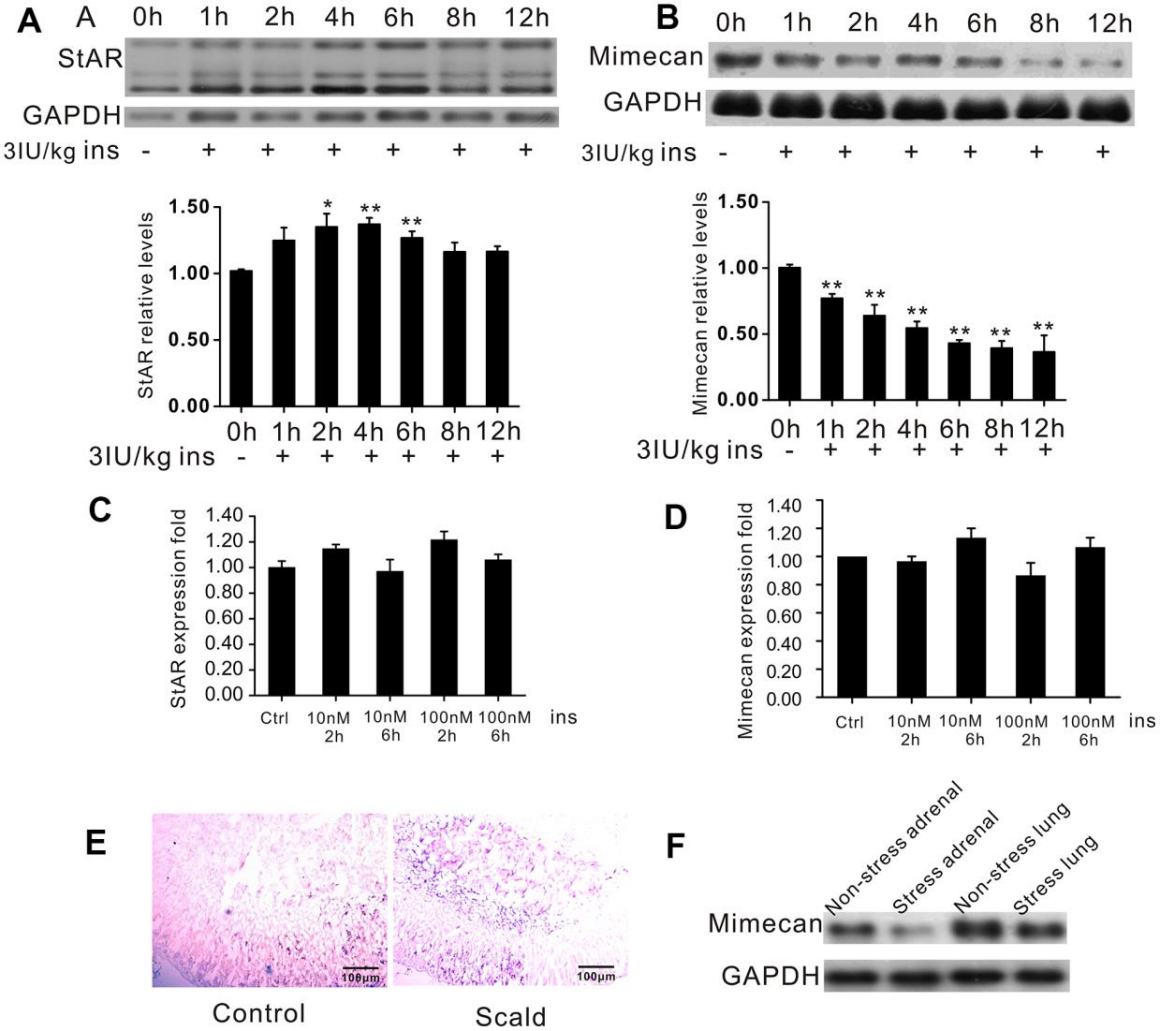
**Downregulation of mimecan expression in the adrenal glands during stress.** (**A**) Northern blot analysis and quantified results confirming the expected increase in StAR gene expression in C57BL/6 male mice subjected to hypoglycemic stress and time-dependent reduction of mimecan gene expression in the adrenal glands of C57BL/6 male mice subjected to hypoglycemic stress (10 mice for each time point). (**B**) Mice were fasted for 12 h and injected with insulin (3 IU/kg). The 0 h group was not injected to avoid stress and served as a control. All other groups were compared with the 0 h group. (**C**, **D**) StAR and Mimecan mRNA levels in Y1 cells after insulin treatment detected by Realtime PCR showed no change compared with the control group that received no treatment. (**E**) Sections of adrenal glands hybridized with fluorescein-labeled StAR antisense probes. Positive signals (blue) were significantly increased compared with the sham operation control group (image obtained under visible light). (**F**) Analysis of mimecan expression by Northern blot analysis in scalded C57BL/6 male mice (15 mice per group), showing pronounced downregulation of mimecan mRNA in adrenal glands and no change in lung tissues. Data information: Data are expressed as means ±SEM. *p<0.05, **p<0.01 for stress treatment vs. control or 0 h group, Student’s t-test.

Hypoglycemia is a physiological stressor that leads to activation of the HPA axis [13]. We established insulin-induced hypoglycemia in mice, a commonly used acute physical stress model, to investigate the expression of mimecan after acute stress [14–18]. The test mice were fasted for 12 h overnight and then given insulin (3 IU/kg body weight) by IP injection. Blood glucose was subsequently measured at various time points. Blood glucose levels were all below 2.2 mmol/L, showing a successfully induced hypoglycemia state in the stressed group (Table 1). As anticipated, the expression of StAR, which is a marker of stress, was induced by hypoglycemic stress, showing significant upregulation at 1 h after insulin dosing and peaking at 6 h (Figure 2A). Interestingly, significant time-dependent downregulation of mimecan expression was observed (Figure 2B). However, blood glucose normalized 8 h after insulin administration (Table 1), implying that reduced mimecan expression is not induced by hypoglycemia state. To exclude the possibility that StAR up-regulation and mimecan down-regulation are induced by insulin itself, rather than by insulin-induced hypoglycemia stress, we examined the StAR and mimecan mRNA levels after insulin stimulation in Y1 cells (Figure 2C, 2D). No significant changes in StAR or mimecan mRNA levels were observed at 2 and 6 h after insulin stimulation. To further verify this result, we established another acute stress model, burn trauma in mice, as described [19]. *In situ* hybridization showed that the expression level of StAR mRNA significantly increased in the adrenal tissues of mice after scalding (Figure 2E), so this stress model appears to be sound. Northern blot analysis showed a significant downregulation of mimecan in adrenal tissue, whereas levels in mouse lung tissues were unchanged after scalding (Figure 2F). The above results reveal that mimecan expression in adrenal tissue is significantly decreased after exposure to acute stress.

**Table 1:** Blood glucose measurement during hypoglycemic stress.

**Group**	**Mean value of blood glucose at 0h** **(mmol/L)**	**Mean value of blood glucose at sacrifice** **(mmol/L)**
control	/	9.35±0.88
case-1h	4.64±0.78	1.78±0.34
case-2h	4.75±0.49	1.46±0.37
case-4h	4.87±0.46	1.77±0.48
case-6h	5.63±0.40	3.67±0.51
case-8h	5.36±0.55	5.23±0.62
case-12h	4.36±0.72	6.20±0.48

### Stress-related mimecan downregulation was mediated directly by ACTH rather than by ACTH-induced glucocorticoid secretion

**Figure 3:**
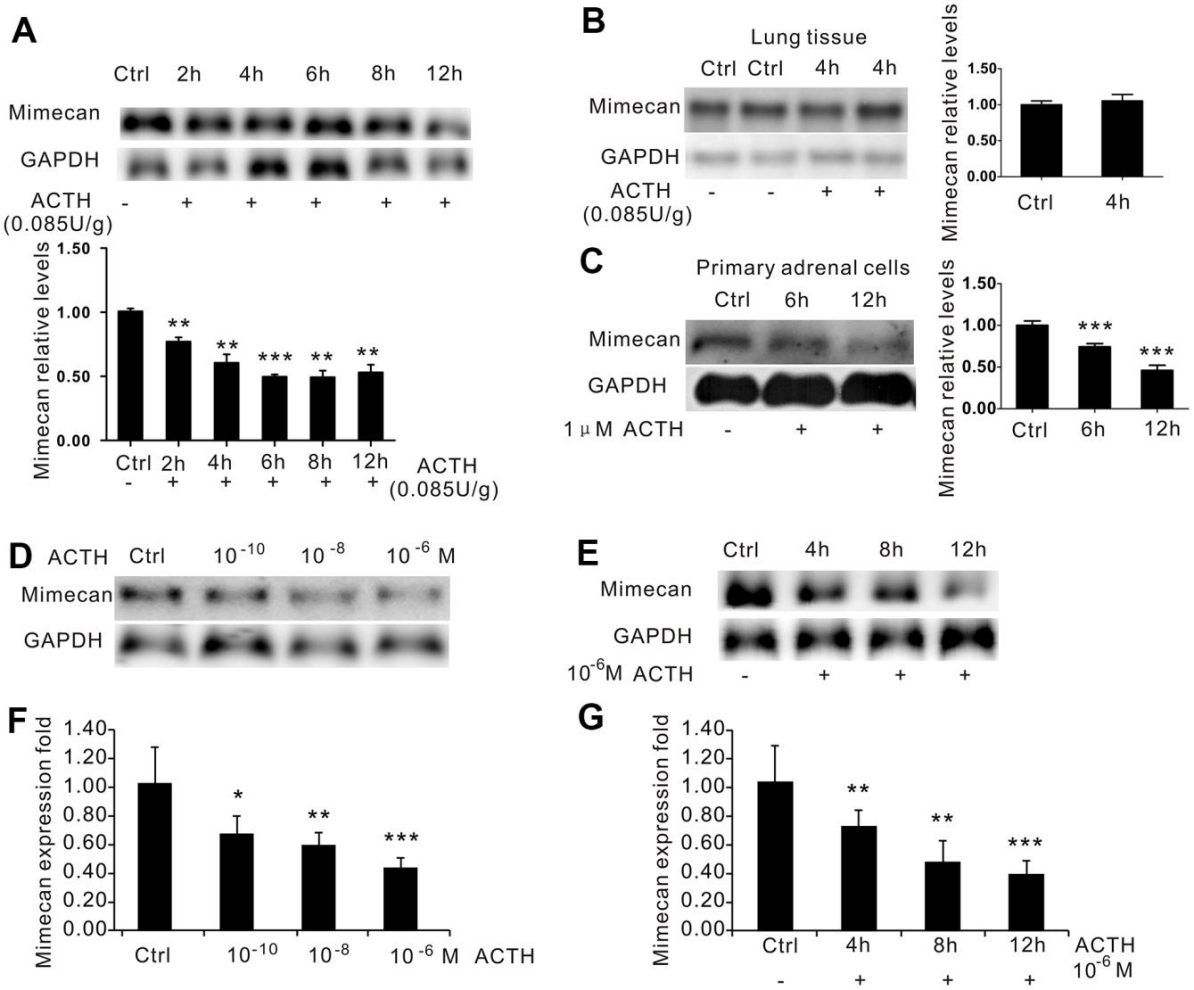
**Inhibition of mimecan expression in time- and dose-dependent manners by ACTH administration.** (**A**) Mimecan mRNA expression was detected by Northern blot analysis in the adrenal glands of C57BL/6 male mice after intraperitoneal injection of adrenocorticotropic hormone (ACTH; 0.085 U/g; 12 mice per group for each time point), showing the significant time-dependent decline in the ACTH injecting group compared with the control group that was not injected. (**B**) No change in mimecan expression was observed in the lung tissue after ACTH dosing. (**C**) Northern blot analysis showing that the expression of mimecan in cultured primary adrenal cells decreased at various time points after the administration of 1 μM ACTH (6 or 7 mice for each time point). (**D**, **E**) Northern blot analysis showing ACTH-induced dose-dependent (**D**) and time-dependent (**E**) inhibition of mimecan mRNA in Y-1 cells. The ACTH dose in (**E**) was 10^-6^ M. Y-1 cells were serum-deprived overnight before the addition of ACTH. (**F**, **G**) Realtime PCR showing ACTH-induced dose-dependent (**F**) and time-dependent (**G**) inhibition of mimecan mRNA in Y-1 cells. Relative mimecan mRNA levels were normalized to GAPDH mRNA expression and compared with untreated controls. Data information: *p<0.05, **p<0.01, ***p<0.001 for ACTH treatment vs. control, Student’s t-test.

Activation of the HPA axis and the accompanying hormonal response to stress is triggered by a surge of CRH into the hypothalamic–pituitary–portal system. Elevated CRH in the portal blood increases ACTH secretion in the pituitary and produces a corresponding rise in adrenal glucocorticoid secretion [20]. To determine whether decreased mimecan expression after stress is mediated by increased ACTH secretion, we administrated an 0.085 U/g dose of ACTH to C57BL/6 mice at 2, 4, 6, 8, and 12 h. The animals were then sacrificed at various time points, and their adrenal glands were collected bilaterally at once using Northern blot analysis to sequentially assess mimecan mRNA expression in the adrenal tissues of these mice. The stated levels showed significant time-dependent declines after ACTH dosing (Figure 3A). Compared with controls, the expression of mimecan in adrenal tissues was reduced by ~70% at 2 h and ~90% at 6 h post-treatment (Figure 3A), whereas the corresponding levels in lung tissue were unchanged (Figure 3B). We also detected mimecan expression after ACTH treatment in primary cultures of mouse adrenal cells (Figure 3C). Similarly, fresh adrenal cellular isolates cultured with 1 μM ACTH for 6 and 12 h revealed declines in mimecan mRNA levels compared with controls (Figure 3C). Considering the complexity of mice, we again applied this strategy to the Y1 adrenocortical cell line. The cells were treated with 10^-10^, 10^-8^, and 10^-6^ M ACTH for 12 h, assessing mimecan mRNA levels by Northern blot analysis (Figure 3D) and real-time quantitative PCR (Figure 3F). Mimecan expression in Y1 cells was downregulated by ACTH treatment in a dose-dependent manner. Compared with controls, the expression of mimecan in the Y1 cell line was reduced by 40% at 10^-8^ M and 60% at 10^-6^ M after ACTH exposure for 12 h (Figure 3D, 3F), and mimecan mRNA levels declined in a time-dependent manner after 1 μM of ACTH treatment, as shown by Northern blot analysis (Figure 3E) and real-time quantitative PCR (Figure 3G).

**Figure 4:**
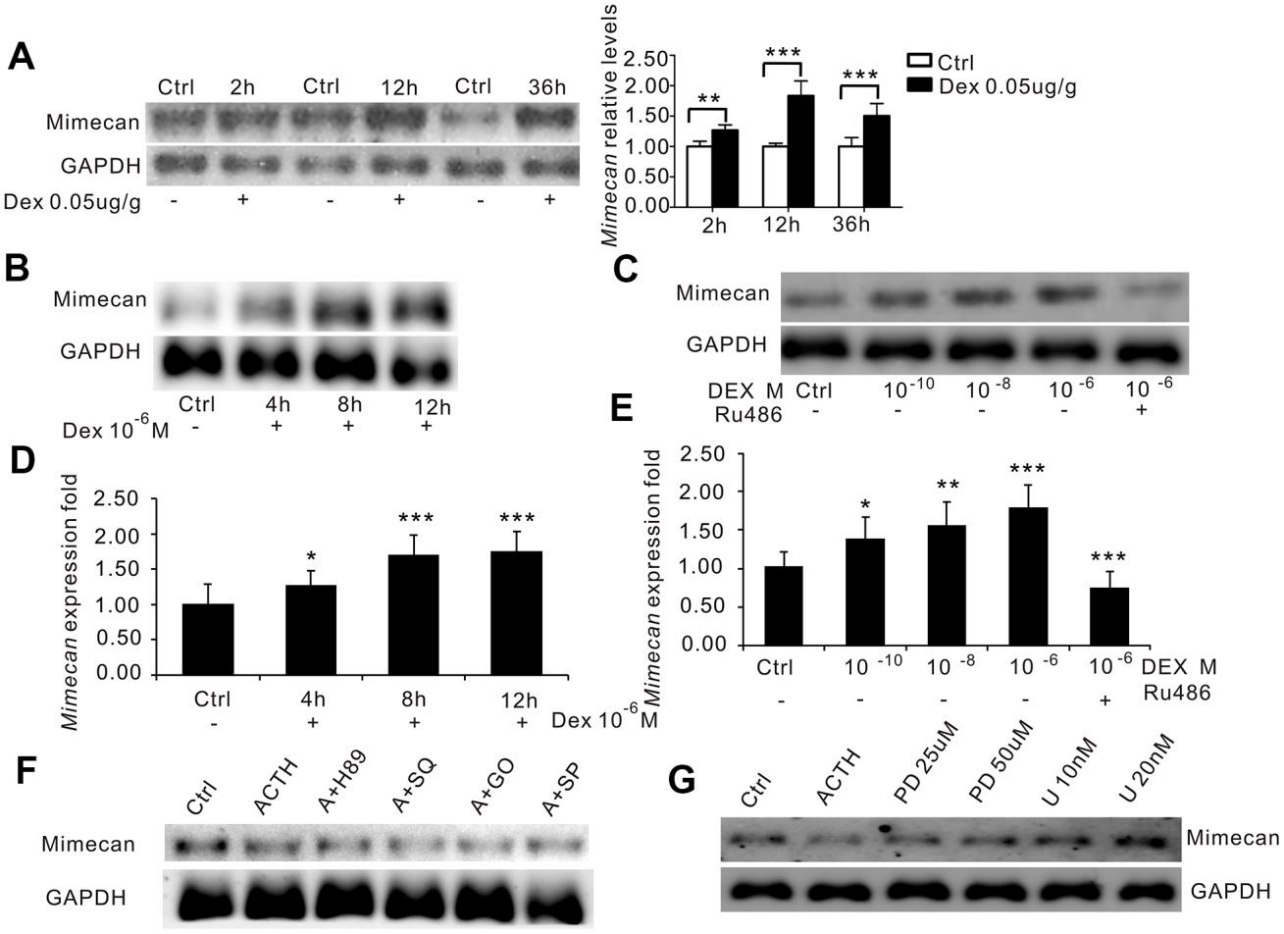
**Dexamethasone (DEX) increases mimecan expression in mice and Y-1 cells.** (**A**) Northern blot analysis reflecting the time-dependent increase in mimecan mRNA expression in the adrenal tissues of the C57BL/6 male mice after intramuscular injection of DEX compared with the corresponding control groups that were injected with 0.9% saline (0.05 μg/g; 10 mice per group for each time point). (**B**) The time-dependent increase in mimecan mRNA in Y-1 cells after DEX treatment (10^−6^ M), analyzed using Northern blot analysis. Y-1 cells were serum-deprived overnight before adding DEX or DEX + RU486. (**C**) The dose-dependent increase in mimecan mRNA in Y-1 cells after DEX treatment was abolished by 1 μM RU486, analyzed using Northern blot analysis. (**D**, **E**) The mRNA levels in (**B**) and (**C**) were determined using quantitative real-time PCR. Relative mimecan mRNA levels were normalized to GAPDH mRNA expression and compared with untreated controls. (**F**) ACTH-induced suppression of mimecan expression cannot be attributed to PKA, cAMP, PKC, or JNK signaling. (**G**) The inhibitory effect of ACTH was abolished by the ERK pathway inhibitors PD98059 and U0126, which rescued mimecan expression in a dose-dependent manner. H89: inhibitor of the PKA pathway; SQ: SQ22536, inhibitor of the cAMP pathway; G0: G06983, inhibitor of the PKC pathway; SP: SP600125, inhibitor of the JNK pathway; PD: PD98059, inhibitor of the ERK pathway; U: U0126, inhibitor of the ERK pathway. Y-1 cells were serum-deprived overnight prior to adding inhibitors. Mimecan gene expression in Y-1 cells was analyzed by Northern blot analysis. The relative mimecan mRNA levels were normalized to GAPDH mRNA expression. Data information: *p<0.05, **p<0.01, ***p<0.001 for DEX treatment vs. control, Student’s t-test.

**Figure 5:**
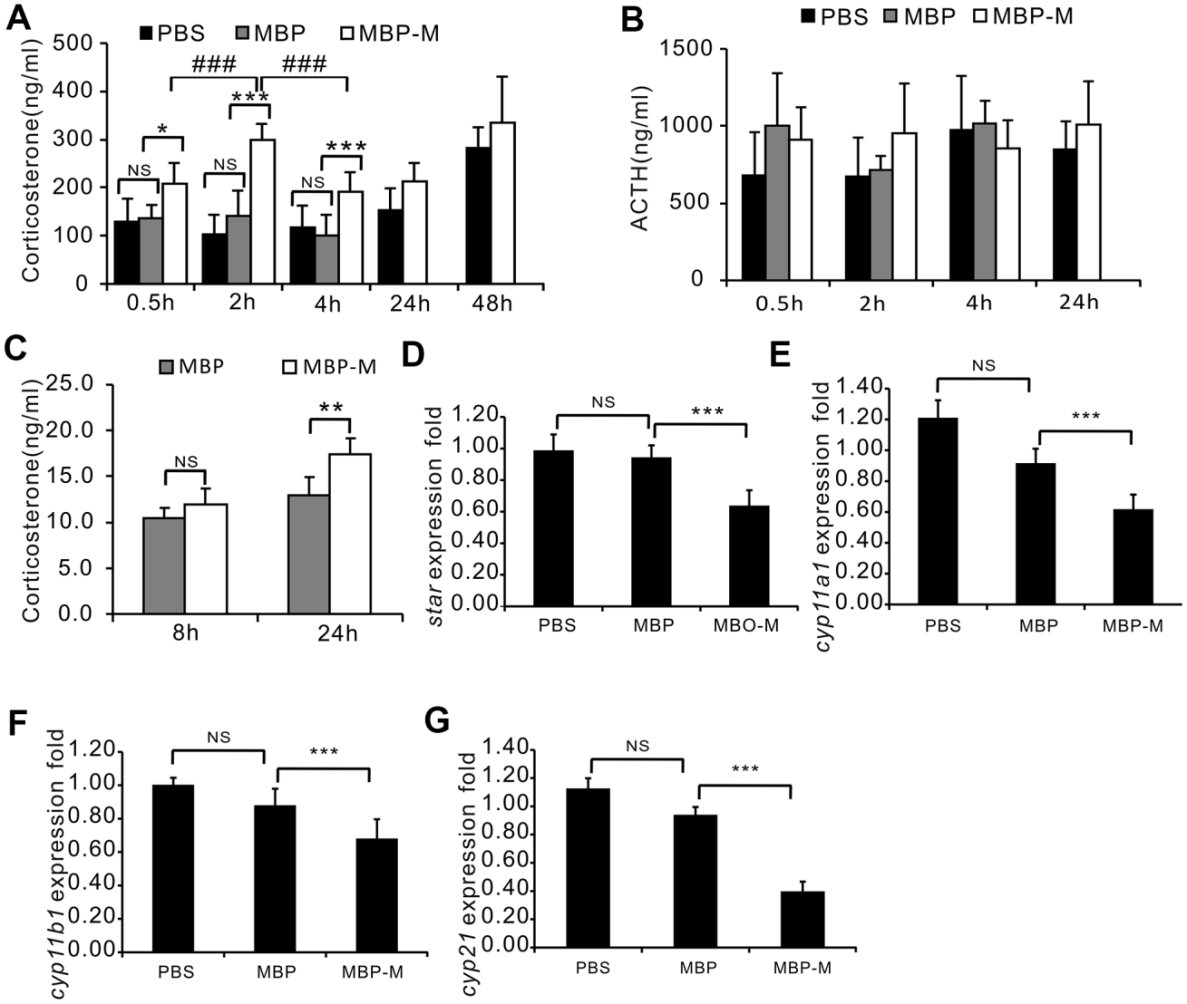
**Mimecan increases corticosterone concentrations *in vivo* and *in vitro* with no effect on corticosterone synthesis.** (**A**) Intraperitoneal injection of recombinant mimecan-MBP (vs. MBP) increases the serum concentration of corticosterone, normalized for 24 h after injection (n=8 per group) in three independent experiments. ***p<0.001 for mimecan-MBP vs. MBP treatment, ### *p*<0.001 for mimecan-MBP treatment at 0.5 h or 4 h vs. 2 h. There was no significant difference between the PBS and MBP injected groups. (**B**) The serum ACTH level was unaffected by intraperitoneal injection of recombinant mimecan-MBP compared with MBP injected controls. Serum corticosterone and ACTH were measured using ELISA (8 mice per group). C57BL/6 mice received intraperitoneal injections of recombinant mimecan-MBP (0.1 μmol/kg), MBP (0.1 μmol/kg), or PBS equivalent (controls). There was no significant difference between the PBS and MBP injected groups. (**C**) Recombinant mimecan-MBP increased the corticosterone concentration in culture supernatant of Y1 cells after 24 h of treatment at 7.64 μM compared with MBP in three independent experiments. (**D**–**G**) mRNA levels of key genes regulating corticosterone synthesis in adrenal tissues declined after intraperitoneal injection of recombinant mimecan-MBP (0.1 μmol/kg) vs. MBP (8 mice per group). There was no significant difference between the PBS and MBP injected groups. Data information: **p<0.01, ***p<0.001 for mimecan-MBP vs. MBP treatment, Student’s t-test.

Because our previous study found that glucocorticoids upregulate mimecan expression in corticotroph cells [11], we also examined the effects of DEX on mimecan expression in adrenal tissues. In accordance with the results in corticotrophin cells, mRNA levels of mimecan in the adrenal tissues of C57BL/6 mice were markedly upregulated in a time-dependent manner after IM injection of DEX (0.05 ug/g body weight), which peaked at 12 h and was sustained for 36 h (Figure 4A). Likewise, mimecan expression in Y-1 cells increased significantly in time- (Figure 4B, 4D) and dose-dependent manners (Figure 4C, 4E) after DEX exposure. Moreover, the DEX-induced increase in Y1 cellular expression of mimecan was abolished by treatment with 1 μM RU486, a GR blocker (Figure 4C, 4E). The results seem contradictory but may be explained by the conjecture that ACTH inhibits mimecan to prevent excessive secretion of glucocorticoids after acute stress, because we found that mimecan also promotes glucocorticoid secretion, as shown in Figure 5A. Moreover, the fact that ACTH lowered mimecan expression and DEX increased its expression during *in vivo* and *in vitro* studies clearly indicates that the effects of stress on the downregulation of mimecan expression in adrenal tissues are mediated by ACTH rather than glucocorticoids.

### Mechanism of inhibited adrenal mimecan expression due to ACTH

The binding of ACTH to its receptor generally activates the following four signaling pathways including cAMP/PKA/CREB, MEK/ERK, PKC and JNK. The cAMP/PKA/CREB pathway is responsible for upregulating StAR expression, which is otherwise downregulated by activated MEK/ERK, PKC, or JNK pathways. To investigate how those pathways mediate the effects of ACTH on mimecan expression, Y1 cells were separately treated with specific inhibitors of four ACTH signaling pathways (SQ22536 for cAMP, H89 for PKA, PD98059 and U0126 for ERK, GO6983 for PKC, and SP600125 for JNK) for 40 minutes prior to stimulation with 1 μM of ACTH. As expected, mRNA levels of StAR were significantly upregulated in Y1 cell lines at 6 h and then abolished after treatment with the cAMP/PKA inhibitors H89 (20 μM) and SQ22536 (200 μM) (Supplementary Figure 1A, 1B, 1E). However, the observed lowering of mimecan mRNA in Y1 cells induced by ACTH was not reversed after treatment with the cAMP/PKA pathway inhibitors H89 (20 μM) and SQ22536 (200 μM) (Figure 3F, Supplementary Figure 2A, 2B). Both the PKC inhibitor GO6983 and the JNK inhibitor SP600125 did not reverse the observed lowering of mimecan mRNA in Y1 cells induced by ACTH (Figure 4F, Supplementary Figure 2C, 2D) and the stimulation effects of ACTH on StAR (Supplementary Figure 1C–1E). It is therefore likely that the inhibition of mimecan expression in adrenal cells due to ACTH is not mediated by the cAMP/PKA/CREB, PKC, or JNK pathways. However, the inhibitory effects of ACTH on mimecan expression in Y1 cells after 6 h of stimulation by 1 μM of ACTH was abolished by treatment with the ERK inhibitors PD98059 (25 μM, 50 μM) and U0126 (10 nM, 20 nM) (Figure 4G). Consequently, ACTH-induced downregulation of mimecan expression in adrenal cells is likely mediated by the ERK signaling pathway.

### Mimecan stimulates the secretion of corticosterone in mouse adrenal cells

Having shown that mimecan is downregulated by ACTH and that corticosterone increases mimecan expression in adrenal tissues, we further investigated whether mimecan regulates ACTH and corticosterone secretion. C57BL/6 mice received IP injections of mimecan-MBP fusion protein, PBS, or MBP alone at doses of 0.1 μmol/kg body weight. Whole blood was then obtained from retro-orbital spaces for ACTH and corticosterone assay (ELISA). There were no significant differences in the serum corticosterone concentrations between the PBS and MBP injected groups; thus, we just compared the MBP-mimecan and MBP injecting groups. Serum corticosterone concentrations were much higher in C57BL/6 mice that received the mimecan-MBP fusion protein at the stated dose than in recipients of MBP (Figure 5A) and these elevated significantly at 0.5 h after IP injection of mimecan-MBP fusion protein, reaching a peak at 2 h, maintaining high levels for at least 4 h and then normalizing at 24 h (Figure 5A). Serum levels of ACTH did not differ significantly among any of these treatment groups (Figure 5B). These data suggest that mimecan-induced corticosterone secretion is not mediated by heightened levels of ACTH. Compared with MBP-treated Y1 cells, the concentration of corticosterone in culture media clearly increased after treatment with mimecan-MBP fusion protein for 24 h, as opposed to after 8 h at a dose of 7.64 μM (Figure 5C). To determine the mechanism responsible for corticosterone elevation, the expression levels of key genes regulating corticosterone synthesis in adrenal tissues, such as *StAR*, *CYP11A1*, *CYP11B1* and *CYP21*, were detected using real-time PCR. Remarkably, the expression levels of these genes in the adrenal tissues of mice were substantially downregulated at 4 h after IP injection of mimecan-MBP fusion protein (Figure 5D–5G). Therefore, the observed mimecan-induced rise in the corticosterone concentration in mouse serum or Y1-cell culture media was probably due to intensified corticosterone secretion by stimulated adrenal cortical cells rather than increased corticosterone synthesis.

### Mimecan deficiency disturbs stress-free diurnal rhythms of corticosterone secretion, enhancing HPA activation in a mimecan knockout mouse restraint model

**Figure 6:**
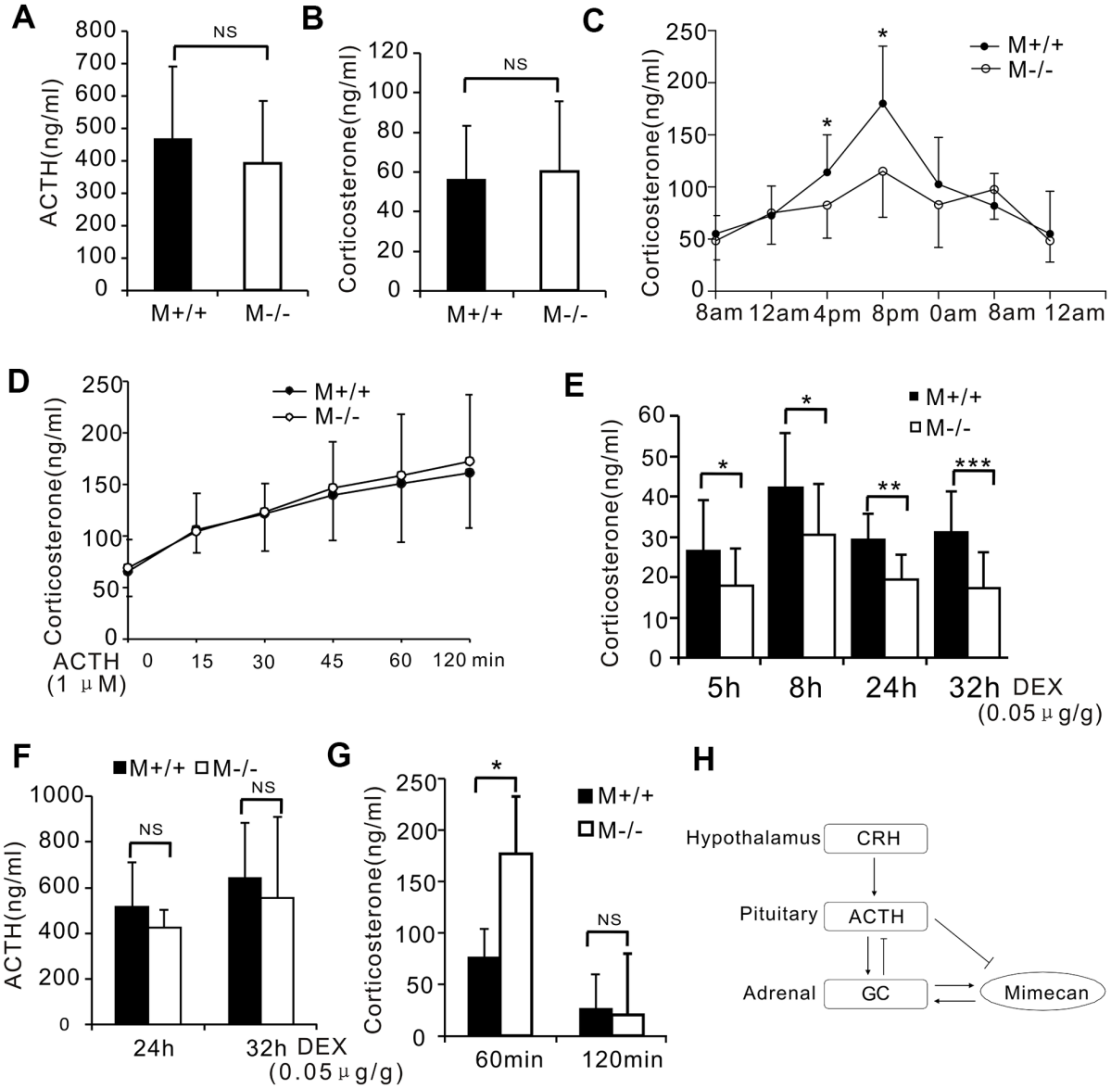
**Disturbed stress-free diurnal rhythm of corticosterone secretion and hyperactivated stress response in mimecan-deficient mice.** (**A**, **B**) No differences in serum corticosterone or plasma ACTH levels of wild-type (WT) and mim^-/-^ mice were determined using ELISA (10 mice per group). (**C**) Significant disturbance of diurnal corticosterone secretion was observed in non-stressed mim^-/-^ mice. Secretion levels lacked the typical peaks and troughs seen with WT mice (n=20 per group). (**D**) There was no difference in the serum corticosterone levels of wild-type (WT) and mim^-/-^ knockout mice after stimulation with 1 μM ACTH. (**E**) The response to the DEX suppression test in mim^-/-^ mice surpassed that of WT mice. The serum corticosterone level was determined using ELISA for 5–32 h after intramuscular injection of 0.05 ug/g DEX (21 mim^-/-^ mice and 15 WT mice). (**F**) Similar serum ACTH levels in mim^-/-^ and WT mice were observed 24–32 h after intramuscular injection of DEX (21 mim^-/-^ mice and 15 WT mice). (**G**) A marked increase in the serum corticosterone level of mim^-/-^ vs WT male mice was observed after 1 h of restraint and this was lost 1 h after release (5 mice per group). Data information: *p<0.05 for mim^-/-^ vs. WT mice, Student’s t-test. (**H**) A graph showing the role of mimecan in the hypothalamic–pituitary–adrenal (HPA) axis.

We further generated mimecan knockout mice to delineate the physiological roles of mimecan. The generation details were described in our previous study [12]*.* The mRNA expression of mimecan was not detected in the adrenal glands of knockout mice (Supplementary Figure 3A). In stress-free states, serum corticosterone and ACTH levels (Figure 6A, 6B) and the mRNA expression level of genes involved in corticosterone synthesis (Supplementary Figure 3B, 3E) were similar for knockout mice and wild-type litter mates. However, the diurnal rhythm of corticosterone secretion in knockout vs. wild-type mice was disturbed significantly, as shown by serial determinations of serum corticosterone collected at 8:00, 12:00, 16:00, 20:00, 0:00, and 4:00 hours in an unstressed state (Figure 6C). In wild-type mice, these levels peaked in the evening (20:00 hours) and reached a nadir in the morning, reflecting non-stressful circadian secretion of cortisol (Figure 6C). However, no clear peaks or troughs of serum corticosterone were encountered in counterpart knockout mice, only a flattened pattern (Figure 6C). To explore the responses of mimecan knockout mice to ACTH and glucocorticoids, these mice received IP injections of ACTH (0.085 IU/g body weight) and IM injections of DEX (0.05 ug/g body weight). Although serum corticosterone levels in knockout and wild-type mice did not differ significantly after ACTH stimulation (Figure 6D), serum corticosterone levels were lower in knockout vs. wild-type mice following IM DEX injection for 5 to 32 h (Figure 6E), which may be due to the absence of corticosterone boosting effects of mimecan in knockout mice. Meanwhile, serum ACTH levels remained comparable in both groups of mice (Figure 6F), and mRNA expression levels of genes encoding corticosterone synthesis enzymes also showed no difference between knockout and wild-type mice after IM DEX injection. (Supplementary Figure 3F, 3I).

We also investigated whether genetic mimecan deficiency influenced pituitary and adrenal gland function in response to stress. Male knockout mice and wild-type litter mates were restrained for 60 min to inflict physical stress. We collected blood through tail cuts at the time of release and 60 min later and then measured serum corticosterone by ELISA. Restraint of knockout mice for 60 min induced a significantly greater increase in serum corticosterone compared with the levels in wild-type litter mates (Figure 6G). Moreover, the observed difference in serum corticosterone concentrations between knockout and wild-type mice was lost at 60 min after release (Figure 6G). These findings indicate a strikingly greater stress response in knockout mice.

## DISCUSSION

According to the present findings, mimecan is primarily expressed in adrenal cortex. We found that its expression in adrenal tissue was significantly downregulated under stress in our mouse model, signifying a role in stress response. We also determined that expression of mimecan in adrenal cells may be downregulated by ACTH and upregulated by DEX. Although both dramatic increases in levels of secreted ACTH and corticosterone were apparent in mice after stress, the data herein clearly illustrate that stress-induced downregulation of mimecan in adrenal tissues is mediated by ACTH, rather than corticosterone. Cortisol is the terminal effector of the HPA axis, and cortisol secretion is stimulated as a result of HPA activation in response to acute stress. It is generally acknowledged that cortisol secreted by the adrenal cortex is directly tied to pituitary ACTH release. In recent years, however, mounting evidence has been collected to show that many adrenomedullary secretory products, such as adrenomedullin, catecholamines, serotonin (5-HT), neuropeptides, and growth factors, take part in regulating adrenocortical steroidogenesis via non-ACTH-dependent pathways [21–23]. Furthermore, cytokines produced by adrenal cells themselves are capable of directly influencing adrenocortical function. They may stimulate steroidogenesis (similar to interleukin (IL)-1, IL-2, and IL-6) or exert regulatory effects on adrenal growth (as does TNFα or interferon-γ) [24–27]. It is a proven fact that cortisol release is self-regulated through a central nervous system (CNS) negative feedback loop involving receptors in pituitary, Paraventricular nucleus, and other brain structures [28]. However, little is known about similar synthesis/secretion autoregulation via intra-adrenal mechanisms.

In the present study, based on *in vivo* and *in vitro* studies, we found that mimecan stimulates corticosterone secretion from adrenal tissues through a short time response, yet there were no *in vivo* effects on the serum levels of ACTH. Additionally, mimecan expression may be upregulated by corticosterone, as shown by DEX administration studies *in vitro* and *in vivo*, and this effect vanished after the treatment of Y-1 adrenal cells with RU486 (a GR inhibitor). This is in accordance with our previous results that the expression of mimecan in pituitary corticotrophin cells is increased by glucocorticoids and that its upregulation may be mediated through classic GR pathways [11]. Our findings therefore suggest that, under basal conditions, mimecan may stimulate glucocorticoid release from adrenal cells as a means of positive feedback (Figure 6H), although this premise is still tentative. Our data support the concept that ACTH-independent glucocorticoid regulation permitting self-regulation of glucocorticoid synthesis and secretion exists within the adrenal glands.

Another interesting facet of this study is that mimecan deficiency in mice fosters disturbances in the diurnal rhythms of corticosterone secretion and stronger responses to stress restraint, despite the similar serum ACTH and corticosterone concentrations seen in wild-type and mimecan knockout mice under basal conditions. Consequently, it is tempting to presume that mimecan plays a vital role in helping to maintain glucocorticoid homeostasis in mice after exposure to acute stress. In basal states of moderate-level ACTH release, glucocorticoids promote mimecan expression in adrenal tissues through GRs, in turn prompting the secretion of glucocorticoids to sustain appropriate levels. Thus, a positive intra-adrenal feedback loop seems to be at work. However, stress-related increases in ACTH release due to HPA axis overactivation will then culminate in the downregulation of intra-adrenal mimecan expression, attenuating the effects of mimecan on glucocorticoid secretion and avoiding excess production of glucocorticoids. Unexpectedly, in mimecan knockout mice, which lack the positive effect of mimecan on corticosterone secretion, there is no change in corticosterone secretion in the basal state, but there is an increase in corticosterone levels under stressful conditions compared with wild-type mice. We hypothesized that other compensatory pathways that are not under negative feedback control by ACTH may be activated in knockout mice to sustain normal corticosterone levels in the basal state, leading to HPA axis overactivation under stressful conditions. The negative feedback effect of mimecan during acute stress is lost in knockout mice, explaining their comparatively stronger responses to stress.

Mimecan is broadly expressed in organs and implicated in many biological processes; thus, we could not determine which process drives the effects of mimecan in the stress response because mimecan may affect any part of HPA axis and even other process that affect the HPA axis. Similarly, we cannot attribute the phenotype of mimecan knockout mice to the loss of mimecan expression in the adrenal tissues. Mimecan is known as an extra cellular matrix protein that helps to maintain matrix homeostasis [29, 30]. In addition, the deficiency or downregulation of mimecan expression has been implicated in several kinds of tumor [31, 32]. The expression of mimecan mRNA and protein has also been observed in the human anterior pituitary glands, and pituitary transcription factor-1 (Pit-1), expressed solely in the pituitary, is capable of activating the human mimecan promoter via Pit-1 response-element sites [33]. Moreover, we discovered that mimecan can inhibit food intake by inducing IL-1β and IL-6 expression in the hypothalamus [10]. Thus, it is possible that mimecan also exerts its functions in the pituitary and hypothalamus.

## CONCLUSIONS

We have demonstrated, for the first time, the critical role that mimecan likely plays in regulating the HPA axis. In the absence of mimecan effects, we observed disturbed corticosterone secretion circadian rhythms in C57BL/6 mice and abnormal secretion of corticosterone in response to stress or exogenous steroid stimulation. Although ACTH may inhibit mimecan expression in adrenal tissues, mechanisms of mimecan control are still unknown. Future studies investigating the impact of mimecan on corticosterone secretion are clearly warranted. More work will be needed to comprehensively explore the functions of mimecan in the stress response.

## MATERIALS AND METHODS

### Animals

Wild-type C57BL/6 mice were bought from Shanghai Experimental Animal Center, Chinese Academy of Sciences. Mimecan knockout mice (mim^-/-^) were generated as described elsewhere [11, 12]. The animals were housed in a temperature-controlled room (23° C) subject to 12-h light/dark cycles and allowed access to chow and water ad libitum. Adult male (8–10 weeks-old) mim^-/-^ mice and age-matched wild-type C57BL/6 mice were used for the experiments. All animal-related investigations were conducted in accordance with our institutional guidelines on ethical animal care and were approved by the Animal Care and Use Committee in the Ninth Hospital Affiliated to Shanghai Jiaotong University School of Medicine.

### Hypoglycemic-mouse model

The hypoglycemic-mouse model was adapted from previous studies [14–17]. C57BL/6 mice were randomly assigned to the stressed or control group at each time point 0, 1, 2, 4, 6, 8, and 12 h (10 mice for each time point). C57BL/6 mice from the stressed group were fasted overnight for 12 h. Blood glucose levels at 0 h (before insulin injection) were assayed in blood obtained by tail cuts at 8 a.m. using a glucometer (Accu-Chek Compact, Roche, Rotkreuz, Switzerland), and intraperitoneal (IP) injections of insulin (3 IU/kg) (Humulin, Lilai, Suzhou, China) were delivered at 9 a.m. [17]. The mice were subsequently sacrificed by cervical dislocation after blood glucose measurement in the tail vein at 1, 2, 4, 6, 8, and 12 h after injection, and adrenal glands and lung tissues were immediately taken and frozen at –80° C. The dosage of insulin was tested to reach a target blood glucose concentration of lower than 2.2 mmol/L, a value conventionally used to define hypoglycemia. Control groups were not injected and were sacrificed 6h after the start of the experiment.

### Scalded-mouse model

The scalded-mouse model was adapted from Liang et al. [19]. C57BL/6 mice were randomly assigned to the scalded or control group (15 mice per group). Twenty-four hours after the hairs of mice were removed by using hair removal agents made from barium sulfide, mice were randomly divided into the stressed group and control group. Both groups of mice were anesthetized with a 35 mg/kg dose of 2.5% pentobarbital sodium and placed on boards designed for 10% total body surface area exposure and immersed (8 seconds) in a water bath held at 90° C (stress group) or room temperature (control group) [19]. This method delivered a full-thickness cutaneous burn, as confirmed by histological examination. Four hours after burn or sham injury, the mice had fully recovered from anesthesia and were sacrificed by cervical dislocation. Unilateral portions of scalded skin were fixed in buffered formalin for morphological evaluation. Part of the samples of adrenal glands and lung tissue were formalin-fixed and paraffin-embedded, and part were immediately frozen and stored at –80° C until analysis.

### Restraint stress model

Five mim^-/-^ mice and five wild-type litter mates were restrained using plastic tubes with a narrow end that exposed the mouse heads, obtaining blood (by tail cuts) after 60 min of restraint and being released for 60 min.

### ACTH stimulation test

The dosage and administration method was based on reported concentrations of ACTH under stress and methods used in previous studies [34–36]. C57BL/6 mice were randomly assigned to each group (12 mice for each time point). We administered ACTH (0.085 IU/g body weight) in mice by IP injection (9:00 a.m.). Then, the mice were sacrificed by cervical dislocation at the indicated time points: 2, 4, 6, 8, and 12 h. Adrenal glands were immediately taken and frozen at –80° C. ACTH (A0673, Sigma-Aldrich, St. Louis, MO, USA) was diluted by sterile 0.9% saline and placed on ice so that it remained effective. Control groups were not injected and were sacrificed 6h after the start of the experiment. In mim^-/-^ mice and wild-type litter mates (21 mim^-/-^ mice and 15 WT mice), the same dose of ACTH was given by IP injection at 9 a.m. Blood samples for corticosterone measurement were removed from the tail vein before and at 15, 30, 45, 60, and 120 min after ACTH administration.

### Dexamethasone suppression test

The dosage and administration method used were based on previous studies [37, 38]. C57BL/6 mice were randomly assigned to each group (10 mice per group for each time point). Dexamethasone (D4902, Sigma-Aldrich, St. Louis, MO, USA) (0.05 ug/g body weight) or 0.9% saline was administered by intramuscular (IM) injection to experimental and control groups, respectively, and mice were sacrificed by cervical dislocation at the indicated time points: 2, 12, and 36 h at 7:30 p.m. (when cortisol secretion peaks). Adrenal glands were immediately taken and frozen at –80° C. In mim^-/-^ mice and wild-type litter mates (21 mim^-/-^ and 15 WT), the same dose of DEX was given by IM injection. Blood samples used for corticosterone and ACTH measurement were removed from the tail vein at 5, 8, 24, and 32 h after DEX administration at 12 a.m.

### Circadian rhythm determination of corticosterone

Eleven mim^-/-^ mice and twelve wild-type litter mates were used, and blood was taken from the tail vein every 4 h for serial serum corticosterone determination.

### Mimecan-MBP stimulation

C57BL/6 mice were administered with 0.1 μmol/kg of mimecan-MBP, 0.1 μmol/kg of MBP, which were made in our lab [10], or an equivalent volume of 0.9% saline by IP injection at 9 a.m. Mice were sacrificed by decapitation, and trunk blood was obtained at 0.5, 2, 24, and 48 h after injection for hormonal analysis. For the 24 and 48 h groups, the injection was repeated every 8 hours.

### Primary adrenal gland cell isolation and cell culture

Primary adrenal gland cells were isolated by the collagenase digestion method. Adrenal glands were obtained from 20 adult male C57BL/6 mice after decapitation. Tissues were washed with HBSS. Pieced, sliced fragments were digested in prepared D-Hanks buffer containing 30 mg/ml of type I collagenase (C0130, Sigma-Aldrich, St. Louis, MO, USA) for 90 min in a 37° C water bath. Dispersed cells were centrifuged and resuspended in F-12K medium containing 15% horse serum and 2.5% fetal calf serum. Then, cells were distributed in 12-well plates and incubated at 37° C under 5% CO_2_ for 12 h until use.

The Y-1 mouse adrenocortical tumor cell line was obtained from the American Tissue Type Collection (ATCC, ATCC, VA, USA), which is a subclone of the corticotropin-responsive cell line originally developed by Yasumura et al. [39]. The cell line was maintained in F-12K medium containing 15% horse serum and 2.5% fetal calf serum (FCS) (GIB-CO, USA) in a 5% CO_2_-humidified atmosphere at 37° C. All cell cultures were routinely passaged at 90–95% confluency. Before the experiment, cells were preincubated with F-12K medium containing 0.2% BSA for 24 h and then treated with ACTH (A0673, Sigma-Aldrich, St. Louis, MO, USA), DEX (D4902, Sigma-Aldrich, St. Louis, MO, USA), MBP-mimecan fusion protein made in our lab [10], specific cell pathway inhibitors, and so on. Medium samples were collected and stored at –80° C at the end of the experiments for hormone content analysis. Cells in the culture plates were processed for RNA extraction, as indicated below.

### Quantitative real-time polymerase chain reaction (PCR)

Gene expression was assessed by relative quantification (2^ΔΔCt^ method), using an ABI Prism 7300 Real-Time PCR System (Applied Biosystems, Foster City, CA, USA), 96-well plates, and SYBR Premix Ex Taq (Takara Bio, Shiga, Japan), in accordance with the manufacturer’s instructions [40]. All samples were normalized to values of β-actin. Results are expressed as fold-changes in the threshold cycle (CT) values relative to controls. Cycling parameters were 95° C for 10 s, and then 40 cycles of 95° C for 5 s and 60° C for 31 s. Analysis was done in quadruplicate, with experiments being independently repeated three times. Primers are shown in Supplementary Table 1.

### Blood collection and hormone assays

Whole blood was collected into iced empty or heparinized tubes. Blood was centrifuged at 2000 g for 20 min at 4° C, and then plasma or serum was recentrifuged at 6000 g for 10 min at 4° C before being stored at –80° C for subsequent determination of ACTH in the plasma and corticosterone in the serum. ACTH or corticosterone concentrations of the mouse plasma or serum and Y-1 cell culture media were measured by commercially available ELISA kits (EK-001-21, Phoenix Pharmaceuticals, USA and Cayman, USA), as described by the manufacturer.

### Fusion protein purification and antibody production

Antibody production of the mimecan-MBP fusion protein and purified MBP protein was conducted as described earlier by our group [10]. The cDNA encoding 12 kDa human mimecan (residues 175–279) was subcloned into pGEX-5X-2 (GE Healthcare, Madison, WI, USA) and overexpressed in *Escherichia coli* BL21 (DE3) cells. Purified mimecan-MBP fusion protein was used for antibody production. Rabbits and mice were immunized with recombinant protein in Freund's adjuvant (Sigma-Aldrich, St. Louis, MO, USA) for polyclonal and monoclonal antibody production. Antibodies were purified using Protein G (GE Healthcare, Madison, WI, USA). The monoclonal subtype was identified as IgG1-κ. For fusion protein purification, human cDNA encoding 12 kDa mimecan (residues 175–279) was subcloned into pMAL-c2x (NEB) and overexpressed in BL21 (DE3) cells. Cells were grown at 37° C to an optical density at 595 nm (A595) of 0.6–0.8, induced with 0.5 mM isopropyl-β-D-thiogalactoside (IPTG) for 5 h, and centrifuged. Cells were sonicated and centrifuged, and the fusion protein in the supernatant was purified by affinity chromatography (MBPTrap HP, GE Healthcare, Madison, WI, USA), gel filtration (Superdex 200, 10/300 GL, GE Healthcare, Madison, WI, USA), and ion exchange (HiTrap ANX FF, GE Healthcare, Madison, WI, USA) chromatography. The purity of the mimecan-MBP fusion protein (58 kDa) was 96%, as determined by sodium dodecyl sulfate polyacrylamide gel electrophoresis (SDS-PAGE) and silver staining. MBP (46 kDa) was expressed and purified (98%) for use as a control.

### Northern blot analysis

Northern blot analysis was performed using the non-isotopic digoxigenin (DIG) Northern Starter Kit (Roche Diagnostics, Rotkreuz, Switzerland), as directed by the manufacturer [33]. Target fragments, mouse mimecan, and mouse steroidogenic acute regulatory protein (StAR) were cloned into PGEM-T Easy vectors and confirmed by restriction enzyme digestion and sequence analysis. DIG-labeled probes were generated by transcription, using SP6/T7 RNA polymerase from the DIG RNA Labeling Kit. Total RNA was isolated from mouse tissues by TRIzol reagent (Invitrogen, Carlsbad, CA, USA), and spectrophotometry was used to gauge the total RNA content. Ten micrograms of mRNA composed of equal amounts of mRNA from 10 mice in a group per lane was applied to a 1.2% agarose–formaldehyde denaturing gel and transferred by capillary blotting to positively charged nylon membranes (Roche, Rotkreuz, Switzerland). The membranes were then baked at 80° C for 2 h. Hybridization was performed at 68° C with overnight agitation. The membranes were washed twice for 5 min (room temperature), using 2× standard saline citrate (SSC) and 0.1% SDS, and then they were washed twice for 15 min (68° C) using 0.1× SSC and 0.1% SDS. Finally, the membranes were washed, blocked, and incubated with anti-DIG serum/alkaline phosphatase conjugate. CDP-Star (Roche, Rotkreuz, Switzerland) served as the chemiluminescence substrate. Signals were visualized on X-ray film.

### *In situ* hybridization

Target fragments (Mimecan) were cloned into a PGEM-T easy vector (A1360, Promega, Madison, WI, USA) and confirmed by automated sequencing. The RNA probes were labeled using the DIG or Fluorescein RNA labeling kit (SP6/T7; Roche, Rotkreuz, Switzerland). The adrenal glands from the C57BL/6 mice were cut into serial frozen sections (5 um). These sections were first fixed in 4% paraformaldehyde and digested in 1 ug/ml of protein kinase buffer. After prehybridization, the sections were incubated with hybridization solution containing 0.5 ng/ul of Fluorescein-labelled probe (for single *in situ* hybridization) or DIG/Fluorescein-labelled probes (for double *in situ* hybridization) in a humidified chamber overnight at 68° C. The post-hybridization slides were washed twice with 2 X SSCT (0.3 M sodium chloride, 30 mM sodium citrate, 0.1% Tween 20)/50% formamide at 68° C and once with 2X SSCT and 0.2X SSCT at room temperature and then incubated with Anti-Fluorescein AP-conjugate (for single) or anti-DIG-alkaline phosphatase Fab (for double) diluted 1:1000 in blocking solution. After being washed in MABT (0.1 M maleic acid, 0.15 M sodium chloride, 0.1 M Tris-base, 0.1% Tween 20, pH 7.5), they were incubated with staining buffer in a humidified chamber. To terminate the reaction, samples were rinsed several times with nuclease-free water and visualized by light microscopy.

For double-staining, phenylethanolamine-N methyl transferase (PNMT), tyrosine hydroxylase (TH), or Adrenomedullin (AM) were visualized by the phosphatase substrate BCIP/NBT, according to the protocol used for single-staining, followed by washing twice with MAB (0.1 M maleic acid, 0.15 M sodium chloride, 0.1 M Tris-base, pH 7.5) for 20 minutes at room temperature. Then, they were incubated with MAB added to 10 mM EDTA for 30 minutes at 65° C to destroy residual anti-DIG-alkaline phosphatase Fab activity.

After blocking for 1 h, sections were incubated overnight at 4° C with Anti-Fluorescein AP-conjugated secondary antibody. After being washed in MABT, they were incubated with Fast red staining buffer in a humidified chamber. To terminate the reaction, samples were rinsed several times with nuclease-free water and visualized by light microscopy or fluorescence microscopy (Leica).

### Immunohistochemical analysis

Sections of formalin-fixed, paraffin-embedded adrenal tissue (4-μm thick) from C57BL/6 mice were rehydrated. Following microwave antigen retrieval, the polyclonal anti-mimecan antibody (1000-fold dilution) was applied for immunostaining. anti-mimecan antibody was generated in our lab by immunizing rabbits with glutathione-S-transferase-mimecan fusion protein, as detailed elsewhere [10]. Chromogenic reactions were peroxidase-based, relying on the EnVision+ system (Dako [Agilent], Santa Clara, CA, USA) and a nuclear counterstain (Gill’s hematoxylin; Thermo Shandon, Pittsburgh, PA, USA). Pre-immune rabbit serum (1000-fold dilution) was applied to adjacent negative control sections.

### Statistical analysis

All data are individually expressed as the mean ± SEM. When statistical analyses were performed, data were compared using Student’s t-tests with the statistical significance set at *p*<0.05.

### Ethics approval and consent to participate

The animal study was performed using protocols approved by the Animal Care and Use Committee in the Ninth Hospital Affiliated to Shanghai Jiaotong University School of Medicine.

### Availability of data and materials

The datasets used and/or analyzed during the current study are available from the corresponding author on reasonable request.

## Supplementary Material

Supplementary Figures

Supplementary Table 1
