# Supplementary material for: The expression of mimecan in adrenal tissue plays a role in an organism’s responses to stress
Source: Aging (Albany NY). 2021 May 10;13(9):13087–107. doi: 10.18632/aging.202991 (PMC8148509; doi:10.18632/aging.202991)
Supplement: Supplementary Figures [file aging-13-202991-s001.pdf]

SUPPLEMENTARY FIGURES

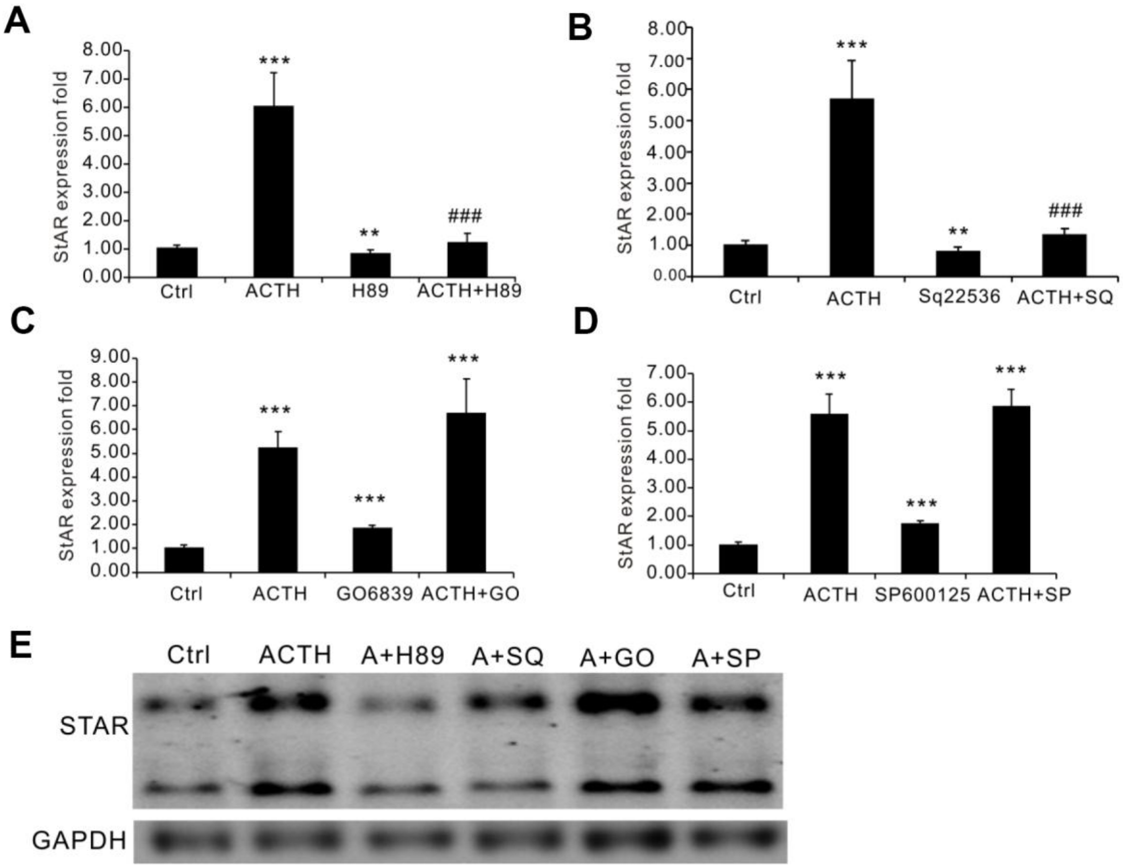

Supplementary Figure 1. StAR mRNA levels in Y1 cells after stimulation by ACTH, inhibitors of related signaling pathways, or both. (**A**, **B**) Significant upregulation of the StAR mRNA expression level was observed in Y1 cell lines after ACTH stimulation (1 μM) for 6 h, and this was abolished by treatment with the cAMP/PKA inhibitors H89 (20 μM) or SQ22536 (200 μM). (**C**, **D**) Significant upregulation of the StAR mRNA expression level was observed in Y1 cell lines after ACTH stimulation (1 μM) for 6 h, but this was not reversed by treatment with the (**C**) PKC inhibitor GO6893 or (**D**) JNK inhibitor SP600125. (**E**) Northern blot analysis confirmed the upregulation of the StAR mRNA expression level in Y1 cell lines after ACTH stimulation (1 μM) for 6 h, and this was abolished by treatment with the cAMP/PKA inhibitors H89 (20 μM) or SQ22536 (200 μM), but it was not reversed by treatment with the PKC inhibitor GO6893 or the JNK inhibitor SP600125. Data information: ***p*<0.01, ****p*<0.001 for ACTH or inhibitor treatment vs. control, ### *p*<0.001 for ACTH and inhibitor treatment vs. ACTH treatment alone, Student's t-test.

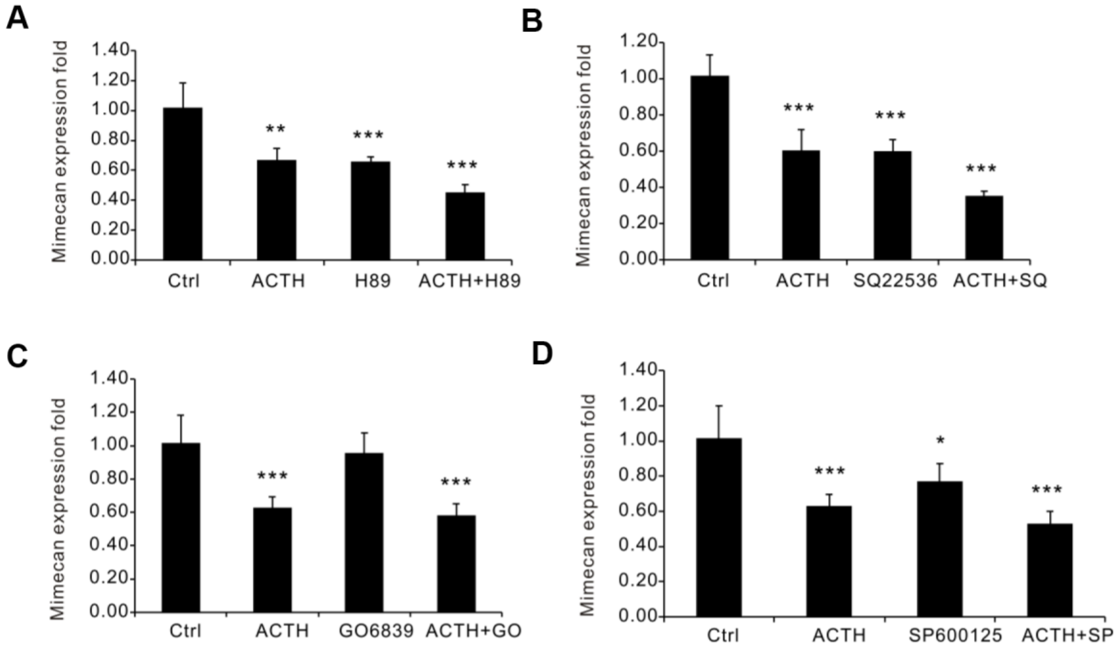

Supplementary Figure 2. Mimecan mRNA levels in Y1 cells after stimulation by ACTH, inhibitors of related signaling pathways, or both. (**A**–**D**) Significant downregulation of mimecan mRNA expression level was observed in Y1 cell lines after ACTH stimulation (1 µM) for 6 h, but this was not reversed by treatment with the (**A**, **B**) cAMP/PKA inhibitors H89 (20 µM) or SQ22536 (200 µM), (**C**) the PKC inhibitor GO6893, or (**D**) the JNK inhibitor SP600125.Data information: *p<0.05, **p<0.01, ***p<0.001 for ACTH or inhibitor treatment vs. control, Student's t-test.

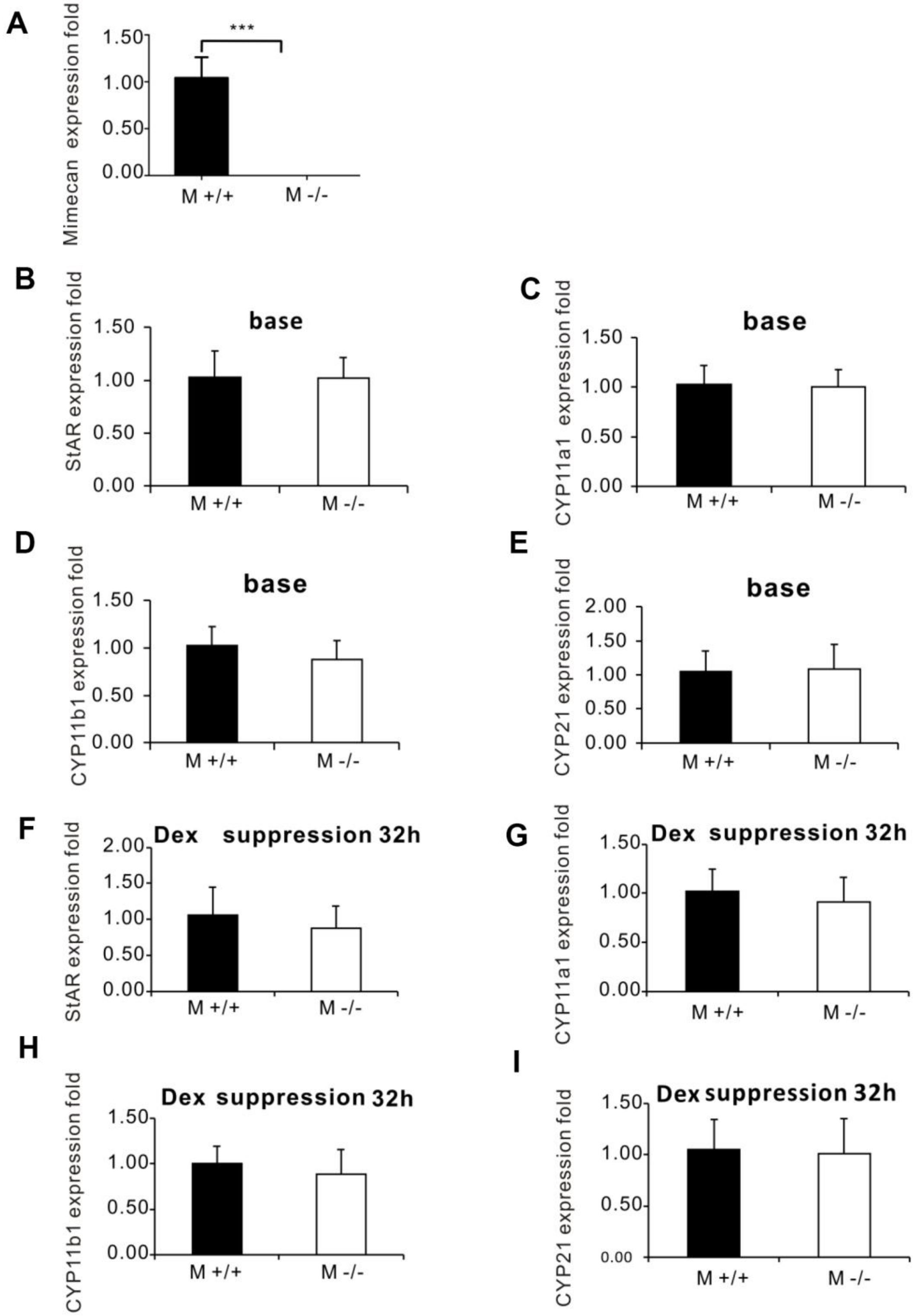

Supplementary Figure 3. The mRNA expression levels of corticosterone synthesis enzymes were unchanged during the base state and DEX suppression test in mim$^{-/-}$ mice compared with WT mice. (**A**) Comparison of mRNA level of mimecan in the adrenal glands. the cycle threshold value was more than 30 in knockout mice. (**B**–**E**) mRNA levels of StAR(**B**), CYP11A1(**C**), CYP11B1(**D**), and CYP21(**E**) were not different between mim$^{-/-}$ and WT mice at baseline. (**F**–**I**) mRNA levels of StAR(**F**), CYP11A1(**G**), CYP11B1(**H**), and CYP21(**I**) were not different between mim$^{-/-}$ and WT mice at 32 h after intramuscular injection of DEX. Data information: **p*<0.05, ***p*<0.01, ****p*<0.001 for mim$^{-/-}$ vs. WT mice, Student's t-test.
