# Supplementary material for: The expression of mimecan in adrenal tissue plays a role in an organism’s responses to stress
Source: Aging (Albany NY). 2021 May 10;13(9):13087–107. doi: 10.18632/aging.202991 (PMC8148509; doi:10.18632/aging.202991)
Supplement: Supplementary Table 1 [file aging-13-202991-s002.pdf]

SUPPLEMENTARY TABLE

Supplementary Table 1. Primer sequences used in quantitative real-time PCR.

Gene	Forward primer	Reverse primer
β-actin	CCTGCTTCAGACCTCCATAGATG	GCGAGAGGTCGAGTTTGCAA
cyp11a1	GTCCATCAGCAGTGTTATATTTGGG	GGAACATCTGGTAGACAGCATTGA
cyp11b1	GCAGGGCCAAGAAAACCTA	TTTCCCTACACTGTGCCTGA
cyp21	AGCTGAAGCAGCACAAGGA	TCGTCTTTGCCATCCCTTT
mimecan	AAAGATGAGGCAATAACACC	TCCAAGTAGAGGAAGGTGAG
StAR	AAGGTGTTTCGCTTGGAGGTG	AGACTTGCAGGCTTCCTGTGAG
